# *Oreocharis wumengensis*, a new species of Gesneriaceae from northeastern Yunnan, China

**DOI:** 10.3897/phytokeys.157.33071

**Published:** 2020-08-26

**Authors:** Lei Cai, Fang-Pu Liu, Xiang-Bo Yi, Zhi-Ling Dao

**Affiliations:** 1 Yunnan Key Laboratory for Integrative Conservation of Plant Species with Extremely Small Populations/ Key Laboratory for Plant Diversity and Biogeography of East Asia, Kunming Institute of Botany, Chinese Academy of Sciences, Kunming 650201, Yunnan, China Chinese Academy of Sciences Kunming China; 2 State Key Laboratory of Systematic and Evolutionary Botany, Institute of Botany, Chinese Academy of Sciences, Beijing 100093, China University of Chinese Academy of Sciences Beijing China; 3 University of Chinese Academy of Sciences, Beijing 100049, China Chinese Academy of Sciences Beijing China; 4 Yunnan Wumengshan National Nature Reserve Administration, Zhaotong 657000, Yunnan, China Yunnan Wumengshan National Nature Reserve Administration Zhaotong China

**Keywords:** flora of Yunnan, morphology, new taxon, *
Oreocharis
*, Wumeng Mountain

## Abstract

A new species of Gesneriaceae, *Oreocharis wumengensis* Lei Cai & Z.L.Dao from Wumeng Mountain area, Yanjin County, Yunnan Province, China, is described. The new species is morphologically similar to *O. panzhouensis* Lei Cai, Y.Guo & F.Wen in the shape of corolla, number of stigma and stamens, but it can be easily distinguished from this species by the leaf shape and indumentum characters of leaf blade, calyx and stamens. Detailed descriptions with photographs of the plant and holotype, and comparisons with morphologically similar species, are also provided.

## Introduction

Many genera and species in the family Gesneriaceae have been redefined in the past two decades based on new evidence following the development of molecular phylogenetics (Möller et al. 2011, 2016; Weber et al., 2011a, 2011b). *Oreocharis* Bentham was redefined in 2011 (Möller et al. 2011), and the vast majority associated species of the other 10 genera were merged into the enlarged genera (Chen et al. 2014, Möller et al. 2014), including some new taxa described in recent years (Cai et al. 2015, 2017, 2019; Chen et al. 2017, 2018; Do et al. 2017; Guo et al. 2018; Han et al. 2017; Möller 2015; Möller et al. 2018; Wei et al. 2016; Yang et al. 2018), *Oreochariss.l.* now comprises ca. 120 species, mainly with southern and southwestern Chinese distribution, but with a few species extending into Vietnam, Myanmar, India, Bhutan, Japan and Thailand (Cai et al. 2017, 2019; Chen 2016; Chen et al. 2017, 2018; Do et al. 2017; Guo and Wang 2014; Li and Wang 2004; Möller et al. 2018; Wang et al. 1990, 1998; Xu et al. 2017).

In April 2017, during field investigations in the Wumeng Mountain area (Yanjin County, northeastern Yunnan), an unknown species of Gesneriaceae without flowers was collected and then planted in Kunming Botanical Garden (KBG). In July 2018, we firstly observed flowering plants which were cultivated in KBG, also based on the floral characteristics; we confirmed that it is a member of *Oreochariss.l.* Following a careful review of the relevant herbarium specimens and taxonomic publications of *Oreocharis* from Yunnan and the adjacent regions, we concluded that this species is new to science. *Oreocharis wumengensis* Lei Cai & Z.L.Dao is described for the first time below, and its morphological characters are compared with those of closely related species.

## Material and methods

Samples of the new species were collected from living plants cultivated in KBG, originally introduced from Yanjin County, Yunnan. All available specimens of *Oreochariss.l.* are stored in the herbaria (HITBC, IBK, KUN and PE) and Chinese Virtual Herbarium (http://www.cvh.ac.cn/) in China and Global Plants on JSTOR (https://plants.jstor.org/) were examined. We studied all morphological characters with dissecting microscopes, and described the morphological characters by using the terminology presented by Wang et al. (1990, 1998). The photographs were taken in the field and KBG by the first author.

## Taxonomic treatment

### Oreocharis wumengensis

Taxon classificationPlantaePasseriformesParamythiidae

Lei Cai & Z.L.Dao sp. nov.

B7E9AE9E-9DB4-5B13-8415-850B7FB736C5

urn:lsid:ipni.org:names:77211188-1

Figures 1 , 2

#### Diagnosis.

*Oreocharis wumengensis* resembles *O. panzhouensis* in its floral characteristics, but can easily be distinguished from this species in the brown-pubescent, oblate petiole; the oblong, long elliptic to oblanceolate leaf blade; the glandular pubescent pedicel; the calyx 5-lobed to the base; and the apically coherent anthers.

**Figure 1.**
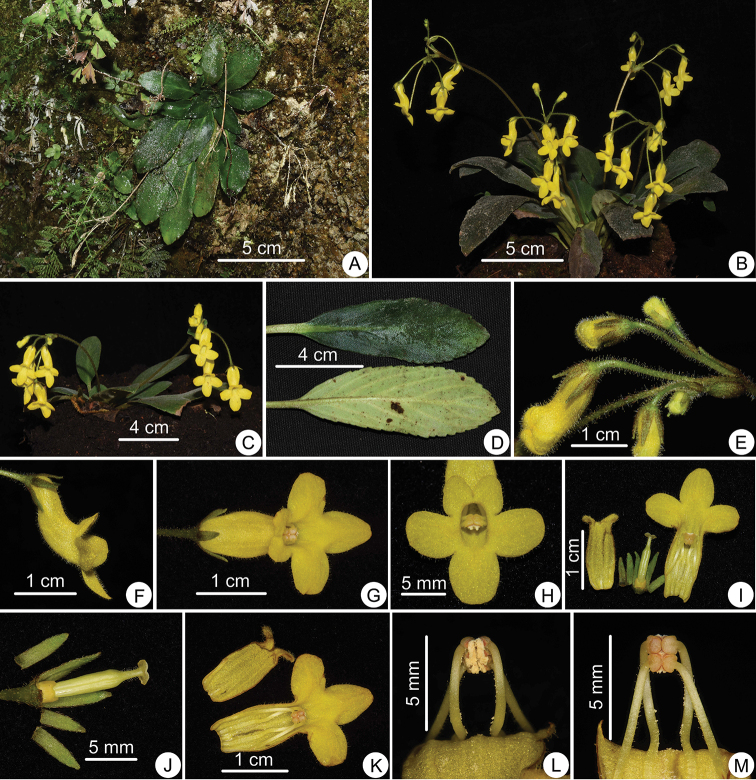
*Oreocharis wumengensis* Lei Cai & Z.L.Dao, sp. nov. **A** Plant in the wild **B, C** plants in cultivation in KBG**D** adaxial and abaxial leaf surfaces **E** inflorescence **F, G** side and top view of flowers **H** front view of flower **I** opened corolla with pistil and calyx **J** pistil with disc and calyx **K** opened corolla showing stamens and staminode **L** adnate anthers, abaxial view **M** adnate anthers, adaxial view. Photographed by Lei Cai.

#### Type.

China. Yunnan: Yanjin County, Miaoba Town, Liuchang Village, Houshanping, 27°52'N, 104°20'E, elev. ca. 1050 m, on moist rocks (cultivated in KBG), in flowering, 3 August 2018, *Lei Cai CL198* (holotype: KUN!, isotype: KUN!).

#### Description.

Perennial herb, rhizome 4–12 mm long, 3–5 mm in diameter. Leaves 8–20, basal, petiole oblate, 0.8–5.5 cm long, brown pubescent, leaf blade oblong, long elliptic to oblanceolate, 3.0–8.5 × 0.8–2.8 cm, adaxially densely appressed pubescent, abaxially pubescent, densely brown pubescent along veins, lateral veins 3–5 on each side of midrib, adaxially inconspicuous, adaxially conspicuous, apex acute, base cuneate, margin serrated, upper half obvious. Cymes axillary 2–4, inflorescence 4–10-flowered; peduncle 6.5–12 cm long, brown pubescent; bracts 2, lanceolate to elliptic, 5–7 × 1.5–3 mm, both sides appressed pubescent, margin nearly entire to denticulate; pedicel 1.5–4.5 cm long, glandular pubescent. Calyx 4–6 mm long, 5-lobed to the base, lobes unequal, linear or narrowly triangular, 4–6 mm long, ca. 1.5 mm wide, outside brown pubescent and glandular pubescent, inside glabrous. Corolla sigmoid, yellow, 2.2–2.6 cm long, outside extremely sparsely brown pubescent and densely glandular pubescent, inside glandular pubescent in the throat and on adaxial lobes, slightly contracted at the throat, 1.2–1.4 cm long, 4–7 mm in diameter; limb 2-lipped; adaxial lip 2-lobed to near base, semiorbicular, lobes 2–3 × 2–3 mm, abaxial lip 3-lobed to base, semiorbicular to oval, 6–8 × 5–7 mm. Stamens 4, 5–8 mm long, adnate to corolla 4–6 mm from base; filaments linear, glandular pubescent; anthers horseshoe shaped, coherent apically, 2-loculed, dehiscing longitudinally, connective glabrous; staminode 1, ca. 0.5 mm long, inserted ca. 1 mm from base. Disc ca. 1.5 mm high, yellow, margin undulate. Pistil 0.8–1 cm long; ovary long cylindrical, glabrous, 4–6 mm long; style 2–3 mm long, glandular pubescent; stigma bilobed, flabellate. Old Capsule linear, ca. 2 cm long.

**Figure 2.**
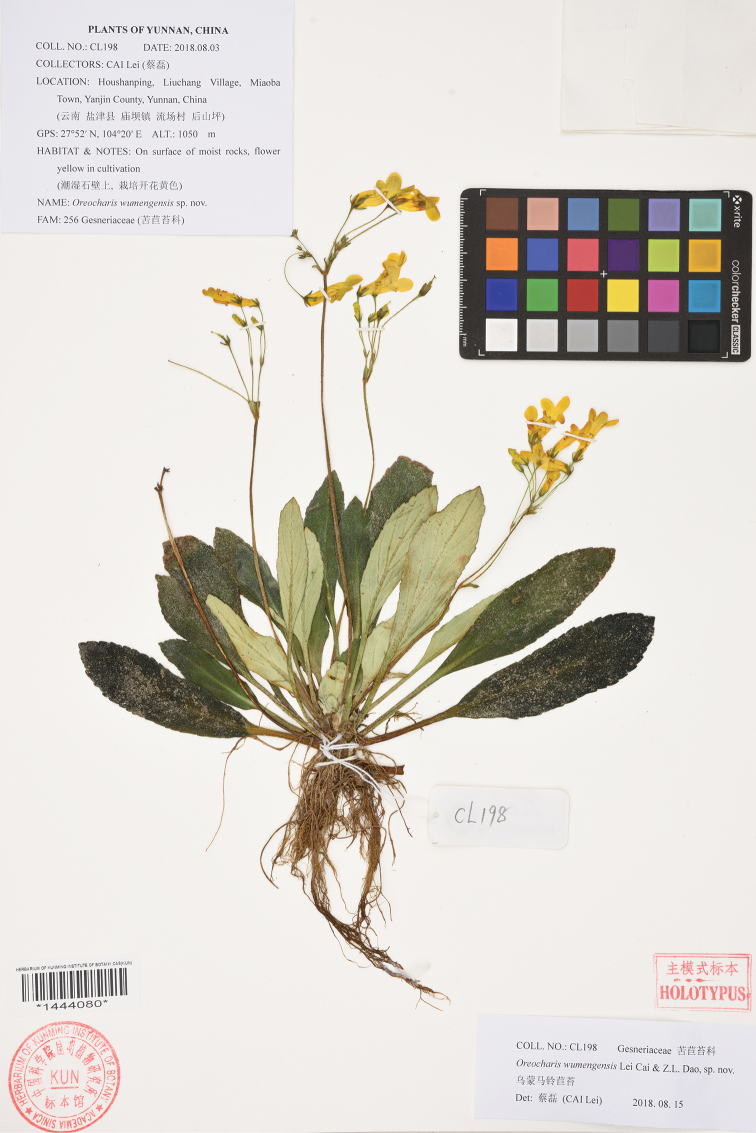
Holotype of *Oreocharis wumengensis* Lei Cai & Z.L.Dao, sp. nov. (KUN-1444080).

#### Phenology.

Flowering from July to August; fruiting unknown.

#### Etymology.

The specific epithet ‘*wumengensis*’ referring to the type locality where the new species was found, is located in the famous Wumeng Mountain area.

#### Vernacular name.

The Chinese name for the new species is “Wū Méng Mǎ Líng Jù Tái” (乌蒙马铃苣苔).

#### Distribution and ecology.

*Oreocharis wumengensis* is currently known only from one population of ca. 50 individuals at the type locality. The species could be endangered, but more data is needed to evaluate this reliably. The species was observed growing on moist rocks with other plants under forest cover in karst regions in Yanjin County, Yunnan.

#### Taxonomic affinities.

*Oreocharis wumengensis* is morphologically unique with sigmoid corolla within *Oreochariss.l.*, however there are certain similarities with other species in this genus. *O. wumengensis* is similar to *O. panzhouensis* in the shape of corolla, however, it is obviously different from the latter species. The comparison of morphologically characters on related species are provided in Table 1.

**Table 1.** Morphological comparison between *Oreocharis wumengensis* and *O. panzhouensis*.

Characters	*O. wumengensis*	*O. panzhouensis*
Petiole	oblate, brown pubescent	round, brown villous
Leaf blade	oblong, long elliptic to oblanceolate	ovate to suborbicular
Peduncle	brown pubescent	brown villous
Bract	lanceolate to elliptic	linear to subulate
Pedicel	glandular pubescent	brown villous
Calyx	5-lobed to the base, lobes linear or narrowly triangular	5-lobed to the middle, lobes broadly triangular
Corolla	sigmoid, yellow, slightly contracted at the throat, outside sparsely brown pubescent and densely glandular pubescent	pale yellow, tube campanulate, outside pubescent and glandular-pubescent
Filament	linear, glandular pubescent	flattened, glabrous
Anthers	horseshoe shaped, coherent apically	broadly oblong, separated
Style	glandular pubescent	glabrous

## Supplementary Material

XML Treatment for Oreocharis wumengensis
